# Somatic mutations in the *p53* gene and prognosis in breast cancer: a meta-analysis

**DOI:** 10.1038/sj.bjc.6690628

**Published:** 1999-08

**Authors:** P D P Pharoah, N E Day, C Caldas

**Affiliations:** CRC Human Cancer Genetics Group and Department of Oncology, University of Cambridge, Strangeways Research Laboratories, Worts Causeway, Cambridge, CB1 8RN, UK; University of Cambridge, Institute of Public Health, Forvie Site, Robinson Way, Cambridge, CB2 2SR, UK; Department of Oncology and Cambridge Institute for Medical Research, University of Cambridge, Wellcome Trust/MRC Building, Cambridge, CB2 2XY, UK

**Keywords:** *p53*, breast cancer, prognosis

## Abstract

Many studies have investigated the association between alterations in the *p53* gene and clinical outcome of breast cancer, and most investigators have reported poorer overall and disease-free survival (as indicated by a relative hazard (RH) greater than one) in breast cancer cases with somatic mutations in *p53*. However, different studies have produced widely differing RH estimates, ranging from no risk (RH = 1) to a relative hazard of 23, and not all of these results have been statistically significant. We have therefore reviewed all the published studies that have investigated the association between somatic mutations in the *p53* gene and breast cancer prognosis and used standard techniques of meta-analysis to combine the results of these studies to produce a more precise estimate of the prognostic significance of *p53* mutations. Eleven studies investigated overall survival in a total of 2319 unselected cases. The RH estimates from these ranged from 1 to 23.4 with a combined RH estimate of 2.0 (confidence interval 1.7–2.5). Three studies investigated the role of *p53* in node-negative patients and in these, the combined estimate of RH was 1.7 (1.2–2.3). For three studies of node-positive breast cancer the combined risk estimate was 2.6 (1.7–3.9). The inclusion of *p53* mutation screening in large breast cancer clinical trials seems warranted in the light of these results. Analysis of large numbers of cases matched for stage and therapy will allow definitive clarification of the value of *p53* mutational status in prognostication, and possibly choice of therapy. © 1999 Cancer Research Campaign


*British Journal of Cancer* (1999) **80**(12), 1968–1973
© 1999 Cancer Research Campaign
Article no. bjoc.1999.0628

### Somatic mutations in the *p53* gene and prognosis in breast cancer: a meta-analysis

**PDP Pharoah[1], NE Day[2] and C Caldas[3]**

[1]CRC Human Cancer Genetics Group and Department of Oncology, University of Cambridge, Strangeways Research Laboratories, Worts Causeway, Cambridge CB1 8RN, UK; [2]University of Cambridge, Institute of Public Health, Forvie Site, Robinson Way, Cambridge CB2 2SR, UK; [3]Department of Oncology and Cambridge Institute for Medical Research, University of Cambridge, Wellcome Trust/MRC Building, Cambridge CB2 2XY, UK

**Summary** Many studies have investigated the association between alterations in the *p53* gene and clinical outcome of breast cancer, and most investigators have reported poorer overall and disease-free survival (as indicated by a relative hazard (RH) greater than one) in breast cancer cases with somatic mutations in *p53*. However, different studies have produced widely differing RH estimates, ranging from no risk (RH = 1) to a relative hazard of 23, and not all of these results have been statistically significant. We have therefore reviewed all the published studies that have investigated the association between somatic mutations in the *p53* gene and breast cancer prognosis and used standard techniques of meta-analysis to combine the results of these studies to produce a more precise estimate of the prognostic significance of *p53* mutations. Eleven studies investigated overall survival in a total of 2319 unselected cases. The RH estimates from these ranged from 1 to 23.4 with a combined RH estimate of 2.0 (confidence interval 1.7–2.5). Three studies investigated the role of *p53* in node-negative patients and in these, the combined estimate of RH was 1.7 (1.2–2.3). For three studies of node-positive breast cancer the combined risk estimate was 2.6 (1.7–3.9). The inclusion of *p53* mutation screening in large breast cancer clinical trials seems warranted in the light of these results. Analysis of large numbers of cases matched for stage and therapy will allow definitive clarification of the value of *p53* mutational status in prognostication, and possibly choice of therapy.

**Keywords**: *p53*; breast cancer; prognosis

The past decade has seen intensive efforts to define molecular genetic events in breast cancer and to correlate these events with its clinical behaviour. One of the most extensively studied genes is the tumour suppressor gene *p53*, which encodes a nuclear phosphoprotein with cancer-inhibiting properties. The currently accepted model for the function of the wild-type p53 protein is as a multi-functional transcription factor involved in the control of cell cycle progression, DNA integrity and cell survival in cells exposed to DNA-damaging agents (Lane, 1992). Arrest of cell cycle progression following DNA damage is thought to represent a basic protective mechanism preventing replication of damaged template DNA. Most of the biologically significant mutations impair the ability of *p53* to participate in the maintenance of genomic stability. As a result, tumours lacking normal p53 might be prone to other deleterious mutations and to be more aggressive clinically.

Many studies have investigated the association between breast cancer prognosis and p53 protein expression in tumour cells with conflicting results. Although most studies have shown a poorer prognosis for breast cancers with increased p53 expression (Thor et al, 1992; Allred et al, 1993; Barnes et al, 1993; Silvestrini et al, 1993; Elledge et al, 1994; Stenmark-Askmalm et al, 1994; Beck et al, 1995; Levesque et al, 1998), others have found no difference (Isola et al, 1992; Bianchi et al, 1997) or even improved (Lipponen et al, 1993; Gohring et al, 1995) survival in this group of cancers. The use of immunohistochemistry (IHC) is based on the fact that mis-sense mutations usually result in an increased half-life of the protein product and a consequent accumulation of the mutant p53 protein in the nucleus. However, many antibodies used are unable to discriminate between the wild-type and mutant p53. Moreover, approximately 20% of *p53* mutations result in protein truncation and these will not be identified by IHC, which has been shown to have a sensitivity of 72% and specificity of 92% compared with sequencing of cDNA to detect *p53* mutations (Norberg et al, 1998).

For these reasons, studies of the association between *p53* mutations and outcome in breast cancer should provide a more reliable indication of the prognostic value of alterations in *p53*. As expected, most investigators have reported poorer overall and disease-free survival (as indicated by a relative hazard (RH) greater than one) in breast cancer cases with somatic mutations in *p53*. In a recent review, Hartmann et al (1997) concluded that 'mutations in the *p53* gene predict poor outcome in breast cancer'. However, different studies have produced widely differing RH estimates, ranging from no risk (RH = 1) to a relative hazard of 23 and not all of these results have been statistically significant.

The aim of this report was to identify all the published studies which have investigated the association between somatic mutations in the *p53* gene and breast cancer prognosis, and to use standard techniques of meta-analysis to combine the results of these studies to produce a more precise estimate of the prognostic significance of *p53* mutations.

*Received 19 November 1998*
*Revised 5 February 1999*
*Accepted 20 February 1999*

*Correspondence to:* PDP Pharoah

**Table 1** Results of *p53* mutation testing for individual studies

| Study | Mutation detection method[a] | Case selection | No. of cases | *p53* alternations | Sequencing results | | | | | |
|---|---|---|---|---|---|---|---|---|---|---|
| | | | | ***n* (%)** | **Total** | **MS** | **NS** | **F** | **IF** | **Other** |
| Andersen et al, 1993 | CDGE exons 5–8 | Unselected | 163 | 35(22) | 35 | 27 (77) | 2 (6) | 6 (17) | 0 | 0 |
| Bergh et al, 1995 | Sequencing cDNA | Unselected consecutive series | 312 | 69 (22) | 69 | 45 (65) | 7 (10) | 11 (16) | 6 (9) | 0 |
| | | Node-positive | 97 | 29 (30) | NA | | | | | |
| | | Node-negative | 201 | 36 (18) | NA | | | | | |
| Berns et al, 1998 | SSCP exons 5–8 | Unselected | 222 | 77 (35) | 66 | 54 (78) | 1 (2) | 4 (6) | 0 | 7 (11) |
| Caleffi et al, 1994 | CDGE exons 5–9 | Unselected | 192 | 43 (22) | 21 | 18 (86) | 2 (10) | 0 | 0 | 1 (5) |
| Elledge et al, 1993 | SSCP exons 5–9 | Node-negative | 200 | 28 (14) | 4 | 1 (25) | 0 | 2 (50) | 0 | 1 (25) |
| Falette et al, 1998 | Sequencing exons 2–11 | Node-negative | 113 | 18 (16) | 18 | 18 (100) | 0 | 0 | 0 | 0 |
| Gretarsdottir, 1996 | CDGE exons 5–8 | Unselected | 186 | 30 (16) | 17 | 12 (71) | 1 (6) | 2 (12) | 0 | 1 (6) |
| Iacopetta et al, 1998 | SSCP exons 4–8 | Node-negative | 422 | 75 (18) | NA | | | | | |
| Kovach et al, 1996 | ddF exons 4–10 | Unselected consecutive series | 44 | 13 (30) | 13 | 8 (62) | 0 | 2 (15) | 3 (23) | 1 (8) |
| Riou et al, 1993 | Sequencing | Inflammatory breast cancer | 24 | 9 (38) | 5 (56) | 1 (11) | 1 (11) | 1 (11) | 0 | 1 (11) |
| Saitoh et al, 1994 | ddF exons 2–11 | Unselected | 52 | 21 (39) | 9 (44) | | | | | |
| Seshadri et al, 1996 | SSCP exons 5–6 | Unselected | 727 | 57 (8) | NA | | | | | |
| | | Node-negative | 424 | NA | NA | | | | | |
| | | Node-positive | 303 | NA | NA | | | | | |
| Shiao et al, 1995 | SSCP exons 5–8 | Unselected | 92 | 18 (20) | 18 | 10 (56) | 2 (11) | 2 (11) | 0 | 4 (22) |
| | | White American | 47 | 9 (19) | 9 | 7 (78) | 0 | 1 (11) | 0 | 1 (11) |
| | | Black American | 45 | 9 (20) | 9 | 3 (33) | 2 (22) | 1 (11) | 0 | 3 (33) |
| Soong et al, 1997 | SSCP exons 4–10 | Unselected | 375 | 70 (19) | 21 | 14 (67) | 2 (10) | 5 (23) | 0 | 0 |
| Thorlacius et al, 1995 | CDGE exons 5, 7, 8 | Unselected | 106 | 20 (19) | 20 | 14 (70) | 1 (5) | 5 (25) | 0 | 0 |
| Tsuda, 1998 | SSCP exons 4–8 | Node-positive | 150 | 38 (25) | NA | | | | | |
| Valgardsdottir, 1997 | CDGE exons 5–8 | Unselected | 87 | 14 (17) | 12 | 10 (83) | 1 (8) | 0 | 1 (8) | 0 |

MS, mis-sense; NS, non-sense; F, frameshift; IF, in-frame insertion/deletion; SSCP, single-strand conformation polymorphism; CDGE, constant denaturing gel electrophoresis; ddF, dideoxy fingerprinting.

### METHODS

Studies investigating the role of somatic mutations in *p53* and prognosis in breast cancer were identified using the Medline (National Library of Medicine, Washington, DC, USA) and BIDS databases for 1983 to July 1998 using the search terms 'breast-neoplasms' and 'p53' and 'mutation'. The bibliographies of any studies identified were also hand searched. Eligible studies were those that reported a survival analysis in breast cancer cases that had been tested for the presence of somatic mutations in *p53*. Where a single study had been reported on multiple occasions, only the most recent report or the report with the most complete data was included in the analysis. Studies that only investigated p53 expression were excluded from the analysis.

### Design of meta-analyses

Details of the calculations described below are given in the Appendix. Combined estimates of risk were obtained by calculating a weighted average of the log relative hazard estimates. Most studies report RH estimates adjusted for other prognostic factors in a multivariate analysis. For the meta-analyses, the adjusted values have been used. The 95% confidence intervals (CI) described are either those published, or have been estimated from published *P*-value associated with the RH estimate. Two studies reported that there was no significant association between *p53* mutations and survival, without giving a RH estimate (Caleffi et al, 1994; Gretarsdottir et al, 1996). In both these studies the published survival curves for the two groups (*p53* mutation + and –) were very close, and so a RH of 1 was assigned. For the purpose of the meta-analysis the weight assigned to the log (RH) for these two studies was similar to the weight for other studies of the same size.

### RESULTS

Sixteen eligible studies were identified. Of these, the breast cancer cases were unselected in 12, one was a small study of inflammatory breast carcinoma (Riou et al, 1993), and three included only cases of node-negative breast cancer. The study of node-negative cancer by Iacopetta et al (1998) was a more detailed analysis of a subset of patients included in a larger study first reported by Seshadri et al (1996).

Table 1 shows the results of *p53* mutation testing in the different studies. A variety of techniques were used to identify genetic alterations including single-strand conformation polymorphism (SSCP), constant denaturing gel electrophoresis (CDGE), dideoxy fingerprinting (ddF) and DNA sequencing. The number of alterations identified by each study are shown in Table 1. Alterations were identified in 539 of 2993 cases tested (18%). This is likely to be an underestimate because most studies limited the analysis to exons 5–8. Around 10% of alterations were found to occur outside this region in studies that analysed other exons (see Table 1). For most of the studies where sequencing was not the primary method for identifying mutations, confirmation of some or all of the alterations identified was carried out by sequencing: 319 (59%) of 539 alterations were confirmed by sequencing, of which 232 (73%) were mis-sense mutations, 20 (6%) were non-sense mutations, 40 (13%) were insertions or deletions resulting in a frameshift, and 26 (8%) were other changes including splice site mutations, complex variants, and in-frame deletions/insertions (Table 1).

© 1999 Cancer Research Campaign

**Table 2** Results of survival analyses for individual studies

| Study | | No. of cases (median follow-up in months) | | Relative hazard (95% CI) | | Variables included in multivariate analysis | Comments |
|---|---|---|---|---|---|---|---|
| | | | | Relapse | Death | | |
| Andersen et al, 1993 | Unselected | 163 | (48) | 2.3 (1.2–4.4) | 2.9 (1.2–7.1) | N,T | |
| Bergh et al, 1995 | Unselected consecutive series | 312 | (57) | NA | 2.0 (1.0–3.9) | A, N, T, ER, S, TX | |
| | Node-positive | 97 | | NA | 2.4 (1.1–5.4) | A, N, T, ER, S, TX | |
| | Node-negative | 201 | | NA | 1.1 (NA) | A, N, T, ER, S, TX | |
| Berns et al, 1998 | Unselected | 177 | (115) | 1.6 (1.1–2.4) | 1.5 (0.97–2.2) | A, N, T, ER, M, c-*myc* | |
| Caleffi et al, 1994 | Unselected | 192 | (48) | NA | not significant | Univariate model | |
| Elledge et al, 1993 | Node-negative | 155 | (71) | 2.2 (1.1–4.3) | NA | A, T, ER, PR, S | |
| Falette et al, 1998 | Node-negative | 113 | (105) | NA | 1.81 (0.99–3.30) | A, T, ER, PR, G | |
| Gretarsdottir, 1996 | Unselected | 186 | (120) | 1.0 (0.9–1.4) | 1.0 (0.9–1.4) | Univariate model | 70% of cases in Iceland 1981–1983 |
| Iacopetta et al, 1998 | Node-negative | 422 | (74) | 1.6 (1.1–2.5) | 2.1 (1.3–3.5) | T, ER, HER-2/neu, MIB-1 | Included data from study first reported by Seshadri et al, 1996 |
| Kovach et al, 1996 | Unselected consecutive series | 90 | (24) | 4.7 (1.4–16) | 23.4 (2.4–228) | N, T, ER, PR | Included data from study first reported by Saitoh et al, 1994 |
| Riou et al, 1993 | Inflammatory breast cancer | 24 | (54) | NA | 8.6(1.4–52.5) | Inflammatory symptoms, ER, *p53* expression | |
| Seshadri et al, 1996 | Unselected | 727 | (NA) | 2.3 (1.5–3.5) | 2.4 (1.5–3.8) | N, T, ER, HER-2/neu | |
| | Node-negative | 424 | | 1.9 (1.0–3.4) | 2.0 (1.0–4.0) | T, ER | |
| | Node-positive | 303 | | 2.5 (1.4–4.4) | 2.7 (1.5–5.0) | T, ER | |
| Shiao et al, 1995 | Unselected | 92 | (NA) | NA | NA | | |
| | Whites | 47 | | NA | 5.6 (1.4–23.0) | A, S | |
| | Blacks | 45 | | NA | 0.81 (0.07–5.51) | A, S | |
| Soong et al, 1997 | Unselected | 198 | (57) | NA | 2.5 (1.2–5.2) | N, S, ER | |
| Thorlacius et al, 1995 | Unselected | 106 | (32) | NA | 3.3 (1.6–6.7) | A, N, T | |
| Tsuda, 1998 | Node-positive | 150 | (44) | 1.9 (1.1–3.3) | 2.7 (1.2–5.9) | Univariate | |
| Valgardsdottir, 1997 | Unselected | 81 | (42) | NA | 6.6 (2.1–20.3) | A, T, N | |

A, age; N, nodal status; T, tumour size; ER, oestrogen receptor status; G, histological grade; PR, progesterone receptor status; M, menopausal status; S, S phase index; c-*myc*, c-*myc* amplification; TX, type of therapy.

The results of survival analyses are given in Table 2. The numbers of cases included in these analyses was frequently less than the number tested for mutations, because of incompleteness of data. Median follow-up ranged from 24 to 120 months. Eleven studies investigated overall survival in a total of 2319 unselected cases (Andersen et al, 1993; Caleffi et al, 1994; Bergh et al, 1995; Shiao et al, 1995; Thorlacius et al, 1995; Gretarsdottir et al, 1996; Kovach et al, 1996; Seshadri et al, 1996; Soong et al, 1997; Valgardsdottir et al, 1997; Berns et al, 1998). The RH estimates from these ranged from 1 to 23.4 with a combined RH estimate of 2.0 (CI 1.7–2.5). However, this result needs to be interpreted with some caution as there was evidence for heterogeneity amongst the studies ($\chi^2$ = 23.2, 10 d.f., $P$ = 0.01). Outcome for node-negative breast cancer according to *p53* mutation status was reported in three studies totalling 736 patients (Bergh et al, 1995; Falette et al, 1998; Iacopetta et al, 1998), one of which was a sub-group analysis of an unselected case series (Bergh et al, 1995). The combined estimate of RH for these was 1.7 (1.2–2.3). Three studies of node-positive breast cancer (Bergh et al, 1995; Seshadri et al, 1996; Tsuda et al, 1998), two of which were sub-group analyses, were carried out for 550 node-positive cases with a combined risk estimate of 2.6 (1.7–3.9). Although the overall survival RH was higher in the node-positive than the node-negative cases there was no statistically significant difference between them ($\chi^2$ = 2.79, 1 d.f., $P$ = 0.09). Disease-free survival was investigated in five studies of 790 unselected patients (Andersen et al, 1993; Gretarsdottir et al, 1996; Kovach et al, 1996; Seshadri et al, 1996; Berns et al, 1998). The combined relative hazard was 1.5 (1.1–1.9). Two studies of 612 node-negative cases (Elledge et al, 1993; Iacopetta et al, 1998) had a combined RH of 1.7 (1.2–2.4) for disease-free survival.

**Table 3** *p53* mutations and survival – results of the meta-analyses

| | Total no. of cases | Relative hazard (95%CI) | | *Homogeneity test* | |
|---|---|---|---|---|---|
| | | | | $\chi^2$ (d.f.) | *P*-value |
| *Overall survival* | | | | | |
| Unselected | 2319 | 2.0 | (1.7–2.5) | 23.2 (10) | 0.01 |
| Node-negative | 736 | 1.7 | (1.2–2.3) | 2.63 (2) | 0.27 |
| Node-positive | 550 | 2.6 | (1.7–3.9) | 0.06 (2) | 0.97 |
| *Disease-free survival* | | | | | |
| Unselected | 790 | 1.5 | (1.2–1.9) | 9.2 (4) | 0.06 |
| Node-negative | 612 | 1.7 | (1.2–2.4) | 0.21 (1) | 0.65 |

Several studies have compared the predictive value of *p53* mutations with that of p53 protein expression (Thorlacius et al, 1995; Kovach et al, 1996; Valgardsdottir et al, 1997; Falette et al, 1998; Iacopetta et al, 1998; Norberg et al, 1998). As would be expected, given the shortcomings of immunohistochemical techniques for the detection of abnormal p53 protein products, all but one of these (Tsuda et al, 1998) found that *p53* mutations were of greater prognostic value than p53 expression.

 *© 1999 Cancer Research Campaign*

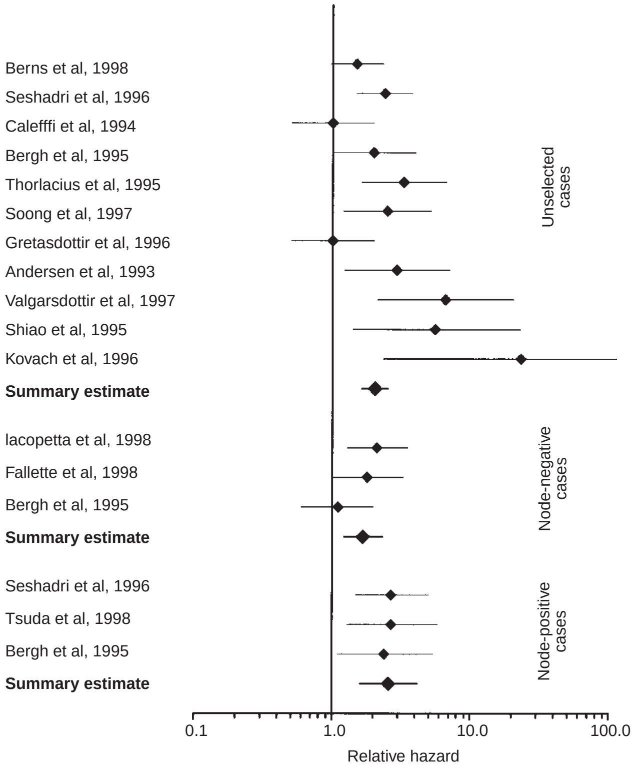

**Figure 1** Funnel plot of relative hazard of overall survival for breast cancer cases with somatic mutation in *p53* by individual study. Studies are plotted in order according to the variance of the log relative hazard estimate. Tendency for smaller studies to have effect sizes greater than the common risk estimate provides evidence for publication bias (see text)

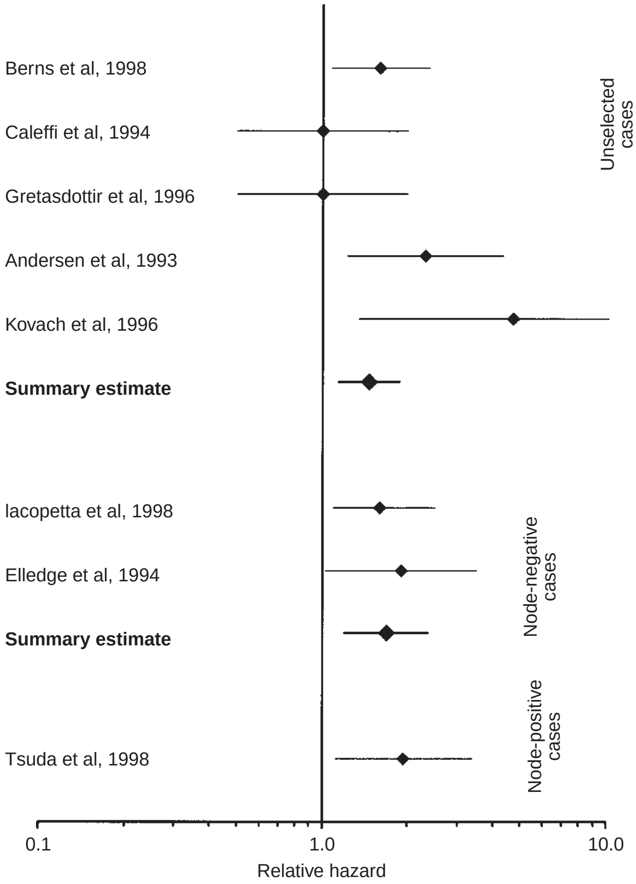

**Figure 2** Funnel plot of relative hazard of disease-free survival for breast cancer cases with somatic mutation in *p53* by individual study

### DISCUSSION

We have identified 16 studies that have investigated the association between somatic mutations in the *p53* gene and survival in breast cancer. The proportion of breast cancers with mutations in *p53* reported in these studies is similar to that from other studies (Hartmann et al, 1997), and the spectrum of mutations is similar to that reported on the *p53* mutations database (International Agency for Research into Cancer, 1998). Greater than 90% mutations reported to this database occur in exons 5–8, and of these, 72% are mis-sense mutations, 7% non-sense, 15% frameshift and 6% other. Most, but not all, studies found that survival was significantly poorer in cancers with a *p53* mutation. In the meta-analysis, the association between *p53* mutation and overall survival was confirmed for unselected, node-negative and node-positive breast cancer. It is possible that this association is the result of confounding by some other factor. However, most studies carried out multivariate analyses to control for a variety of other known prognostic markers, and whichever factors were included in these analyses, *p53* was retained in the final multivariate models. In addition, where the results of both univariate and multivariate analyses were reported, the univariate RHs for *p53* mutations were little different from the multivariate RHs. This suggests that *p53* is an independent prognostic marker.

The possibility of bias also exists, and in interpreting the results of a meta-analysis, three important questions need to be asked:

1. Have all relevant published studies been identified?
2. Are the results of the studies compatible with each other (is there heterogeneity)?
3. Has there been publication bias?

Whether we have been able to ascertain completely all relevant studies is unclear. However, we believe we have identified all published studies in which a survival comparison between breast cancers with and without *p53* mutations was a major component of the study. The importance of possible study heterogeneity is also difficult to assess. Given the differences between studies in study populations, treatment regimens, methods for determining *p53* mutation status, and measurement of potential confounding factors, some degree of heterogeneity between studies is expected. Indeed, for the 11 studies with unselected cases, there was statistical evidence of heterogeneity, with no single study making a substantial individual contribution to the heterogeneity statistic. Whether it is then appropriate to combine the results of these studies depends to some extent on the sources of that heterogeneity. For example, there is some evidence that the prognostic significance of *p53* mutations varies between node-negative and node-positive patients, and so differences in patient populations with respect to node status could account for some of the heterogeneity. However, the effect of this is likely to be limited as, where reported, the proportion of node-negative patients was similar in the various studies. Publication bias (discussed below) is another potential source of heterogeneity, and likely to be more important.

© 1999 Cancer Research Campaign 

The possibility of publication bias – that is the non-publication of studies with findings that are not statistically significant – is a major concern in any systematic review. If publication bias is operating, one would expect that of published studies, the larger ones report the smaller effects. This is because small positive trials are more likely to be published than small negative ones (Egger and Smith, 1995). The occurrence of this can be examined using the funnel plot (Figure 1) in which the effect size is plotted against sample size/variance. In the absence of publication bias, the plot will resemble an inverted funnel centred on the combined risk estimate, with the results of the smaller studies being more widely scattered than those of the larger studies. This, however, does not occur for the 11 studies of unselected cases. The seven larger studies are fairly evenly scattered about the common risk estimate, but for the four smaller studies, the RH estimate increases inversely with study. This suggests, as predicted, that there has been selective publication of small studies with significant positive results. Because these studies are small, they carry less weight than the larger studies, and have only a minor effect on the combined RH estimate. Excluding the four smallest studies from the combined analysis reduces the combined RH estimate from 2.0 (1.7–2.5) to 1.8 (1.4–2.3). Although the observed publication bias will produce an overestimate of the true association, it is extremely unlikely that publication bias has resulted in a Type I error; that is the finding of a significant association, where no such association exists. We estimate that a study or studies of 1500 cases with RH of 0.5 (i.e. in the opposite direction to that expected) would be needed to change the statistically significant RH for overall survival to statistical non-significance.

We have confirmed that, in general, mutations in *p53* confer a worse overall survival and disease-free survival in breast cancer cases, and this effect is independent of other risk factors. Whether the prognostic significance of all mutations is the same is open to doubt. Bergh et al (1995) reported that prognosis for mutations in conserved regions II and V was worse than for mutations in the conserved regions III and IV and non-conserved regions, and Borresen et al (1995) reported that mutations in the zinc-binding domain (Codon 163–195 and 236–251) have worse prognosis than mutations elsewhere.

Doubt also remains about the therapeutic significance of *p53* mutations. One study suggested that locoregional radiotherapy improves survival in breast cancer cases with *p53* mutations but not for those with wild-type *p53* (Jansson et al, 1995). However, another study found that adjuvant systemic therapy, especially with tamoxifen, along with radiotherapy seemed to be of less value to *p53* mutation tumours (Bergh et al, 1995), and Aas et al (1996) found that *p53* mutations were associated with primary resistance to doxorubicin therapy. If these findings were to be confirmed, they would have significant clinical implications.

Answers to questions of the prognostic and therapeutic significance of *p53* status are most likely to be obtained by the inclusion of *p53* mutation screening in large breast cancer clinical trials. Although costly, the cost would be justified by the clinical importance of the questions. Only an analysis of large numbers of cases matched for tumour size and nodal status and therapy will allow definitive clarification of the added value of *p53* mutational status in prognostication, and possibly choice of therapy.

### ACKNOWLEDGEMENTS

PDPP is a CRC Clinical Research Fellow. CC is funded by the CRC.

### REFERENCES

Aas T, Borresen AL, Geisler S, Smith-Sorensen B, Johnsen H, Varhaug JE, Akslen LA and Lonning PE (1996) Specific p53 mutations are associated with de novo resistance to doxorubicin in breast cancer patients. *Nat Med* **2**: 811–814

Allred DC, Clark GM, Elledge R, Fuqua SA, Brown RW, Chamness GC, Osborne CK and McGuire WL (1993) Association of p53 protein expression with tumor cell proliferation rate and clinical outcome in node-negative breast cancer. *J Natl Cancer Inst* **85**: 200–206

Andersen TI, Holm R, Nesland JM, Heimdal KR, Ottestad L and Borresen AL (1993) Prognostic significance of TP53 alterations in breast carcinoma. *Br J Cancer* **68**: 540–548

Barnes DM, Dublin EA, Fisher CJ, Levison DA and Millis RR (1993) Immunohistochemical detection of p53 protein in mammary carcinoma: an important new independent indicator of prognosis? *Hum Pathol* **24**: 469–476

Beck T, Weller EE, Weikel W, Brumm C, Wilkens C and Knapstein PG (1995) Usefulness of immunohistochemical staining for p53 in the prognosis of breast carcinomas: correlations with established prognosis parameters and with the proliferation marker, MIB-1. *Gynaecol Oncol* **57**: 96–104

Bergh J, Norberg T, Sjogren S, Lindgren A and Holmberg L (1995) Complete sequencing of the p53 gene provides prognostic information in breast cancer patients, particularly in relation to adjuvant systemic therapy and radiotherapy. *Nat Med* **1**: 1029–1034

Berns EM, van Staveren IL, Look MP, Smid M, Klijn JG and Foekens JA (1998) Mutations in residues of TP53 that directly contact DNA predict poor outcome in human primary breast cancer. *Br J Cancer* **77**: 1130–1136

Bianchi S, Calzolari A, Vezzosi V, Zampi G, Cardona G, Cataliotti L, Bonardi R and Ciatto S (1997) Lack of prognostic value of p53 protein expression in node-negative breast cancer. *Tumori*, **83**: 669–672

Borresen AL, Andersen TI, Eyfjord JE, Cornelis RS, Thorlacius S, Borg A, Johansson U, Theillet C, Scherneck S, Hartman SI, Cornelisse CJ, Hovig E and Devilee P (1995) TP53 mutations and breast cancer prognosis: particularly poor survival rates for cases with mutations in the zinc-binding domains. *Genes Chrom. Cancer* **14**: 71–75

Breslow NE and Day NE (1980) *Statistical Methods in Cancer Research*, Vol. 1 *The Analysis of Case-Control Studies*. International Agency for Research on Cancer: Lyon

Caleffi M, Teague MW, Jensen RA, Vnencak-Jones CL, Dupont WD and Parl FF (1994) *p53* gene mutations and steroid receptor status in breast cancer. Clinicopathologic correlations and prognostic assessment. *Cancer* **73**: 2147–2156

Egger M and Smith GD (1995) Misleading meta-analysis. *Br Med J* **310**: 752–754

Elledge RM, Fuqua SA, Clark GM, Pujol P, Allred DC and McGuire WL (1993) Prognostic significance of *p53* gene alterations in node-negative breast cancer. *Breast Cancer Res Treat* **26**: 225–235

Elledge RM, Clark GM, Fuqua SA, Yu YY and Allred DC (1994) p53 protein accumulation detected by five different antibodies: relationship to prognosis and heat shock protein 70 in breast cancer. *Cancer Res* **54**: 3752–3757

Falette N, Paperin MP, Treilleux I, Gratadour AC, Peloux N, Mignotte H, Tooke N, Lofman E, Inganas M, Bremond A, Ozturk M and Puisieux A (1998) Prognostic value of P53 gene mutations in a large series of node-negative breast cancer patients. *Cancer Res* **58**: 1451–1455

Gohring UJ, Scharl A, Heckel C, Ahr A and Crombach G (1995) P53 protein in 204 patients with primary breast carcinoma—immunohistochemical detection and clinical value as a prognostic factor. *Arch Gynaecol Obstet* **256**: 139–146

Gretarsdottir S, Tryggvadottir L, Jonasson JG, Sigurdsson H, Olafsdottir K, Agnarsson BA, Ogmundsdottir H and Eyfjord JE (1996) *TP53* mutation analyses on breast carcinomas: a study of paraffin-embedded archival material. *Br J Cancer* **74**: 555–561

Hartmann A, Blaszyk H, Kovach JS and Sommer SS (1997) The molecular epidemiology of *p53* gene mutations in human breast cancer. *Trends Genet* **13**: 27–33

Iacopetta B, Grieu F, Powell B, Soong R, McCaul K and Seshadri R (1998) Analysis of p53 gene mutation by polymerase chain reaction – single strand conformation polymorphism provides independent prognostic information in node-negative breast cancer. *Clin Cancer Res* **4**: 1597–1602

International Agency for Research into Cancer (1998) The IARC database of somatic p53 mutations in human tumors and cell lines. http://www.iarc.fr/p53/homepage.htm.

Isola J, Visakorpi T, Holli K and Kallioniemi OP (1992) Association of overexpression of tumor suppressor protein p53 with rapid cell proliferation and poor prognosis in node-negative breast cancer patients. *J Natl Cancer Inst* **84**: 1109–1114

*© 1999 Cancer Research Campaign*

Jansson T, Inganas M, Sjogren S, Norberg T, Lindgren A, Holmberg L and Bergh J (1995) p53 Status predicts survival in breast cancer patients treated with or without postoperative radiotherapy: a novel hypothesis based on clinical findings. *J Clin Oncol* **13**: 2745–2751

Kovach JS, Hartmann A, Blaszyk H, Cunningham J, Schaid D and Sommer SS (1996) Mutation detection by highly sensitive methods indicates that p53 gene mutations in breast cancer can have important prognostic value. *Proc Natl Acad Sci USA* **93**: 1093–1096

Lane DP (1992) Cancer. p53, guardian of the genome. *Nature*, **358**: 15–16

Levesque MA, Katsaros D, Yu H, Giai M, Genta F, Roagna R, Ponzone R, Massobrio M, Sismondi P and Diamandis EP (1998) Immunofluorometrically determined p53 accumulation as a prognostic indicator in Italian breast cancer patients. *Int J Cancer* **79**: 147–152

Lipponen P, Ji H, Aaltomaa S, Syrjanen S and Syrjanen K (1993) p53 protein expression in breast cancer as related to histopathological characteristics and prognosis *Int J Cancer*, **55**: 51–56

Norberg T, Lennerstrand J, Inganas M and Bergh J (1998) Comparison between p53 protein measurements using the luminometric immunoassay and immunohistochemistry with detection of p53 gene mutations using cDNA sequencing in human breast tumors. *Int J Cancer* **79**: 376–383

Riou G, Le MG, Travagli JP, Levine AJ and Moll UM (1993) Poor prognosis of p53 gene mutation and nuclear overexpression of p53 protein in inflammatory breast carcinoma. *J Natl Cancer Inst* **85**: 1765–1767

Seshadri R, Leong AS, McCaul K, Firgaira FA, Setlur V and Horsfall DJ (1996) Relationship between *p53* gene abnormalities and other tumour characteristics in breast-cancer prognosis. *Int J Cancer* **69**: 135–141

Shiao YH, Chen VW, Scheer WD, Wu XC and Correa P (1995) Racial disparity in the association of *p53* gene alterations with breast cancer survival. *Cancer Res* **55**: 1485–1490

Silvestrini R, Benini E, Daidone MG, Veneroni S, Boracchi P, Cappelletti V, Di Fronzo G and Veronesi U (1993) p53 as an independent prognostic marker in lymph node-negative breast cancer patients. *J Natl Cancer Inst* **85**: 965–970

Soong R, Iacopetta BJ, Harvey JM, Sterrett GF, Dawkins HJ, Hahnel R and Robbins PD (1997) Detection of p53 gene mutation by rapid PCR-SSCP and its association with poor survival in breast cancer. *Int J Cancer* **74**: 642–647

Stenmark-Askmalm M, Stal O, Sullivan S, Ferraud L, Sun XF, Carstensen J and Nordenskjold B (1994) Cellular accumulation of p53 protein: an independent prognostic factor in stage II breast cancer. *Eur J Cancer* **30A**: 175–180

Thor AD, Moore DH II, Edgerton SM, Kawasaki ES, Reihsaus E, Lynch HT, Marcus JN, Schwartz L, Chen LC, Mayall BH and Smith HS (1992) Accumulation of p53 tumor suppressor gene protein: an independent marker of prognosis in breast cancers. *J Natl Cancer Inst* **84**: 845–855

Thorlacius S, Thorgilsson B, Bjornsson J, Tryggvadottir L, Borresen AL, Ogmundsdottir HM and Eyfjord JE (1995) TP53 mutations and abnormal p53 protein staining in breast carcinomas related to prognosis. *Eur J Cancer* **31A**: 1856–1861

Tsuda H, Sakamaki C, Tsugane S, Fukutomi T and Hirohashi S (1998) A prospective study of the significance of gene and chromosome alterations as prognostic indicators of breast cancer patients with lymph node metastases. *Breast Cancer Res Treat* **48**: 21–32

Valgardsdottir R, Tryggvadottir L, Steinarsdottir M, Olafsdottir K, Jonasdottir S, Jonasson JG, Ogmundsdottir HM and Eyfjord JE (1997) Genomic instability and poor prognosis associated with abnormal TP53 in breast carcinomas. Molecular and immunohistochemical analysis. *APMIS* **105**: 121–130

### APPENDIX

### Estimation of 95% confidence intervals (CIs) for relative hazard (RH) where not reported in individual study

$\beta_i$ = In (RH) for individual study

$$SE(\beta_i) = \beta_i / z_{\alpha/2} \quad \text{where } \alpha = \text{p-value}$$
$$UCI(\beta_i), LCI(\beta_i) = \beta_i \pm 1.96 \times SE(\beta_i)$$

Exponentiation of $UCI(\beta_i)$,$LCI(\beta_i)$ gives UCI(RH), LCI(RH).

### Estimation of common RH

The combined estimate of the log RH was estimated from the weighted average of the logarithms of the observed (individual study) RHs (Breslow and Day, 1980).

Let $\beta$ = log(common RH)

$$\text{Then } \beta = \frac{\sum w_i \beta_i}{\sum w_t} \quad \text{with confidence intervals } UCI(\beta), LCI(\beta) = \beta \pm 1.96 \times SE(\beta)$$

Where $w_i$ = weight individual studies = $^1/_{\text{variance}}$

$$\text{variance} = \frac{\log(UCI) - \log(LCI)}{3.92^2}$$

$$SE(\beta) = 1/\sqrt{\sum w_i}$$

The common RH with 95% confidence intervals is then obtained by exponentiation.

### Testing for homogeneity of individual RH estimates

The null hypothesis of homogeneity of individual RH estimates was tested by the $\chi^2$ statistic on $I-1$ degrees of freedom, where I equals number of studies, using the formula

$$\chi^2_{I-1} = \sum w_i \beta^2_i - \frac{\left[\sum w_i \beta_i\right]^2}{\sum w_i}$$

*© 1999 Cancer Research Campaign*
